# Variant spectrum of *PIEZO1* and *KCNN4* in Japanese patients with dehydrated hereditary stomatocytosis

**DOI:** 10.1038/s41439-023-00235-y

**Published:** 2023-03-02

**Authors:** Erina Nakahara, Keiko Shimojima Yamamoto, Hiromi Ogura, Takako Aoki, Taiju Utsugisawa, Kenko Azuma, Hiroyuki Akagawa, Kenichiro Watanabe, Michiko Muraoka, Fumihiko Nakamura, Michi Kamei, Koji Tatebayashi, Jun Shinozuka, Takahisa Yamane, Makoto Hibino, Yoshiya Katsura, Sonoko Nakano-Akamatsu, Norimitsu Kadowaki, Yoshiro Maru, Etsuro Ito, Shouichi Ohga, Hiroshi Yagasaki, Ichiro Morioka, Toshiyuki Yamamoto, Hitoshi Kanno

**Affiliations:** 1 Department of Transfusion Medicine and Cell Processing, Tokyo Women’s Medical University, Tokyo, Japan; 2 Department of Pediatrics and Child Health, Nihon University School of Medicine, Tokyo, Japan; 3 Institute for Comprehensive Medical Sciences, Tokyo Women’s Medical University, Tokyo, Japan; 4 Institute of Medical Genetics, Tokyo Women’s Medical University, Tokyo, Japan; 5 Department of Hematology and Oncology, Shizuoka Children’s Hospital, Shizuoka, Japan; 6 Department of Pediatrics, Fukuyama Medical Center, Okayama, Japan; 7 Department of Laboratory Medicine, Nara Prefecture General Medical Center, Nara, Japan; 8 Department of Pediatrics and Neonatology, Nagoya City University Graduate School of Medical Sciences, Nagoya, Aichi Japan; 9 Department of Neonatology, Gifu Prefectural General Medical Center, Gifu, Japan; 10 Department of Pediatrics, Uji-Tokushukai Medical Center, Kyoto, Japan; 11 Department of Hematology, Osaka City General Hospital, Osaka, Japan; 12 Department of Respiratory Medicine, Shonan Fujisawa Tokushukai Hospital, Fujisawa, Kanagawa Japan; 13 Department of Metabolism and Endocrinology, Tokyo Medical University Ibaraki Medical Center, Ibaraki, Japan; 14 Department of Hematology, Omihachiman Community Medical Center, Shiga, Japan; 15 Department of Internal Medicine, Division of Hematology, Rheumatology and Respiratory Medicine, Faculty of Medicine, Kagawa University, Kagawa, Japan; 16 Department of Pharmacology, Tokyo Women’s Medical University, Tokyo, Japan; 17 Department of Pediatrics, Hirosaki University Graduate School of Medicine, Aomori, Japan; 18 Department of Pediatrics, Kyushu University Graduate School of Medical Sciences, Fukuoka, Japan

**Keywords:** Disease genetics, Disease genetics

## Abstract

Hereditary stomatocytosis (HSt) is a type of congenital hemolytic anemia caused by abnormally increased cation permeability of erythrocyte membranes. Dehydrated HSt (DHSt) is the most common subtype of HSt and is diagnosed based on clinical and laboratory findings related to erythrocytes. *PIEZO1* and *KCNN4* have been recognized as causative genes, and many related variants have been reported. We analyzed the genomic background of 23 patients from 20 Japanese families suspected of having DHSt using a target capture sequence and identified pathogenic/likely pathogenic variants of *PIEZO1* or *KCNN4* in 12 families.

## Hemolytic anemia: new variants in hereditary subtypes

Genetic variants identified in 12 Japanese families are likely to be responsible for a rare hereditary condition causing red blood cells (RBCs) to rupture. Keiko Shimojima Yamamoto of Tokyo Women’s Medical University, Japan, and colleagues conducted genetic analyses on blood samples from 23 people in 20 Japanese families with suspected dehydrated hereditary spherocytosis (DHSt), caused by RBC ion imbalances leading to dehydration and rupture. In 12 families, the analyses revealed probable disease-causing variants in two genes, *PIEZO1* and *KCNN4*, some of which were identified for the first time. Both genes are related to RBC ion channels and the mutations lead to two slightly different forms of DHSt. Further understanding could improve treatment strategies. Comprehensive genomic analysis is a powerful tool for understanding the genetic cause of congenital hemolytic anemia.

## Introduction

Hereditary stomatocytosis (HSt) is a type of congenital hemolytic anemia caused by abnormally increased cation permeability of erythrocyte membranes^1,2^. The most common subtype of HSt (dehydrated HSt [DHSt] or hereditary xerocytosis [HX]) is diagnosed by screening tests, such as evaluation of erythrocyte morphology, measurement of the cation concentration inside and outside the erythrocyte membrane, or osmotic gradient ektacytometry^3^.

DHSt is an autosomal dominant hemolytic anemia characterized by abnormally shaped red blood cells (RBCs) and associated with primary erythrocyte dehydration^4^. DHSt is thought to be rare, and a prevalence estimate of 1:50,000 has been suggested^1^. This condition is characterized by mild to moderate hemolysis with varying numbers of stomatocytes on peripheral blood smears, which are sometimes rare, ill-formed, and likely overlooked. The reticulocyte count is elevated, and the mean cellular hemoglobin concentration (MCHC) and mean cell hemoglobin content (MCH) are increased. Paradoxically, the red cell mean corpuscular volume (MCV) is slightly increased^5^. Patients may also present with a history of perinatal edema and show pseudohyperkalemia due to the loss of potassium ions from RBCs stored at room temperature. Complications such as splenomegaly and cholelithiasis may occur due to increased trapping of RBCs in the spleen and elevated bilirubin levels, respectively. Furthermore, DHSt is frequently associated with iron overload, which may lead to hepatosiderosis^6^, diabetes mellitus, failure of the pituitary gland, and heart failure.

In 2012, DHSt was first identified as being related to alterations in the piezo-type mechanosensitive ion channel component 1 gene (*PIEZO1*; MIM* 611184)^7^. *PIEZO1* encodes a mechanosensitive ion channel that translates a mechanic stimulus into calcium influx^7^. The identified missense variants showed the gain-of-function *PIEZO1* phenotype, providing insight to help explain the increased permeability of cations in RBCs of patients with DHSt^8^. Dehydrated hereditary stomatocytosis 1 with or without pseudohyperkalemia and/or perinatal edema (DHS1: OMIM#194380) is a dominantly inherited red cell membrane disorder caused by gain-of-function mutations of *PIEZO1* in most cases.

In 2015, another gene, the potassium intermediate/small conductance calcium-activated channel, subfamily N, member 4 gene (*KCNN4*; MIM*602754), encoding the calcium ion-dependent potassium selective Gardos channel, was identified as being associated with DHSt^9^. Dehydrated hereditary stomatocytosis-2 (DHS2, OMIM#616689) is caused by a heterozygous mutation in *KCNN4*.

We participated in clinical research on patients with hereditary red cell membrane disorders. For this purpose, we developed a target capture sequencing (TCS) system for precise and comprehensive diagnosis of suspected hereditary red cell membrane disorders in patients^10^. Previously, we reported the genetic background of hereditary spherocytosis, which can be distinguished by morphological characteristics of RBCs and osmotic abnormalities of RBC membranes^10^. Here, we report the genomic variants identified in Japanese patients with HSt, especially those related to DHSt.

## Materials and methods

From April 2015 to June 2021, 20 Japanese families with suspected DHSt were enrolled in this study. This study was performed in accordance with the principles of the Declaration of Helsinki and approved by the ethics committee of the institution. After obtaining written informed consent, blood samples were collected from all patients. In addition, we collected detailed clinical information from the attending doctors, including family histories, clinical courses, and physical findings.

In most patients, when possible, we first performed additional red cell membrane functional examinations, including the acidified glycerol hemolysis time (AGLT) test, flow-cytometric osmotic fragility (FCM-OF) test, and eosin-5’-maleimide (EMA) binding test with a negative direct antiglobulin test as per previously reported methods^10^. DHSt was suspected when clinical findings such as hemolytic anemia with stomatocytosis and hemochromatosis not due to transfusion, positive family history, and past history of perinatal edema were observed, and laboratory tests revealed elevated MCV, increased % residual red cells (%RRC) in the FCM-OF test, and normal or increased EMA binding.

Genomic DNA was extracted from the patient’s peripheral blood using a QIAamp DNA extraction kit according to the manufacturer’s instructions (QIAGEN, Hilden, Germany). The Haloplex HS target enrichment system (Agilent Technologies, Santa Clara, CA, USA) was used for TCS. Using SureDesign (https://earray.chem.agilent.com/suredesign/home.htm), the target panel was designed to include all coding exons and intron‒exon boundaries of the 74 possible candidate genes^10^. Massive parallel sequencing was performed using the Illumina MiSeq platform (Illumina Inc., San Diego, CA, USA). Raw data were aligned to the human genome sequence GRCh37/hg19. The generated FASTQ files were imported into SureCall v3.5 (Agilent Technologies) for variant calling. Analysis following the filtering of the obtained variants was described previously^10^. The obtained variants were filtered according to the following strategy: (1) variant frequencies were below 1% in 1000G_EAS and ALL (1000 Genomes), HGDV, and dbSNP; (2) synonymous variants were excluded (nonsynonymous variants, variants associated with frameshift, insertion/deletion variants, and variants in splicing donor/acceptor sites were included); (3) variants with allele frequencies less than 30% of the total read depth were excluded; and (4) the CADD_phred was higher than 20 if obtained. Variant information obtained using wANNOVAR (http://wannovar.wglab.org/) was used for curation. Integrative Genomics Viewer (https://software.broadinstitute.org/software/igv/) was used for visual evaluation. All variants were evaluated using the guidelines proposed by the American College of Medical Genetics and Genomics and the Association for Molecular Pathology (ACMG/AMP)^11^.

The existence of the identified variants in the probands of the enrolled patients was confirmed using conventional PCR-Sanger sequencing. Genotyping for αLELY (low expression allele of *SPTA1*), *UGT1A1*, and Memphis I and II (*SLC4A1*) was also performed using conventional PCR-Sanger sequencing for all patients^12–15^.

## Results

Among the 20 examined families, 12 were shown to have pathogenic or likely pathogenic variants of *PIEZO1* or *KCNN4* (diagnosis ratio of 60%) in accordance with the ACMG/AMP guidelines^11^. All the variants were confirmed by Sanger sequencing (Supplemental Figs. S1 and S2). The patients’ clinical and genetic information is summarized in Table 1.

**Table 1:** Clinical information and the results of this study.

Patient number		Patient 1	Patient 2	Son of patient 2	Patient 3	Patient 4	Daugther of patient 4	Mother of patient 4	Patient 5	Patient 6	Patient 7	Patient 8	Patient 9	Patient 10	Patient 11	Patient 12
Gender		F	M	M	F	M	F	F	M	F	F	M	F	F	F	M
Age at examination		47 y	39 y	8 m	2 m	68 y	31 y	89 y	69 y	19 y	16 y	28 y	15 y	68 y	14 y	8 y
Family history		−	+	+	−	+	+	+	+	+	−	+	−	NA	+	−
Clinical features																
Splenomegaly		−	−	−	NA	NA	+	−	+	−	−	+	−	+	−	NA
Splenectomy		−	+	−	−	NA	NA	NA	−	−	−	−	−	−	+	−
Gallstone		NA	+	NA	−	NA	NA	NA	+	NA	NA	NA	−	+	NA	+
Previous blood transfusion		−	+	−	+	−	NA	+	+	−	+	+	+	+	+	+
Other findings		Diabetes mellitus, failure of the pituitary gland	Failure of the pituitary gland, Infertility, Heart failure, Cerebral infarction, Renal infarction	Fetal edema	Fetal edema	Diabetes mellitus			Extramedullary hematopoiesis					Diabetes mellitus		
RBC morphology																
Target cell		+	+	+	−	+	NA	NA	+	+	+	+	+	+	+	+
Stomatocyte		+	+	+	−	+	NA	NA	+	+	+	+	+	+	+	−
Acanthocyte		−	+	−	+	−	NA	NA	−	−	−	−	−	−	+	−
Elliptocyte		−	−	−	−	−	NA	NA	−	+	−	−	−	−	−	+
Poikilocyte		−	−	−	−	−	NA	NA	−	−	−	−	−	−	−	+
Schistocyte		−	−	+	−	−	NA	NA	−	−	+	−	−	−	−	−
Anisocyte		−	−	−	+	+	NA	NA	−	−	−	+	+	−	−	−
Polychromasia		−	−	−	+	−	NA	NA	−	−	−	−	−	−	−	−
Nucleated erythrocyte		−	+	−	−	−	NA	NA	−	−	−	−	−	−	−	−
Laboratory findings																
Hb (g/dL)	[13 < : male, 12 < : female]	12.3	8.4	10.0	10.5	14.6	12.7	6.9	13.5	15.6	11.0	10.1	9.7	8.5	10.7	10.1
MCV (fL)	[86–98]	112.3	115.6	88.0	85.0	93.3	91.7	90.8	107.1	92.7	103.6	107.4	107.1	108.3	95.3	86.6
MCHC (%)	[31–35]	34.6	34.4	36.0	34.9	37.6	37.0	39.0	35.8	36.0	38.2	34.8	33.0	34.4	37.7	35.6
Reticulocytes (‰)	[0.2–2.7]	10.4	12.6	8.4	2.5	8.3	12.7	8.5	7.1	13.8	6.7	11.5	8.0	16.7	23.9	6.4
LDH (U/L)	[240–490]	186	284	236	308	202	173	187	157	144	142	175	139	156	191	239
Haptoglobin (mg/dL)	[19–170]	97.5	3.0	NA	NA	86.0	NA	NA	16.0	56.0	13.0	2.0	18.0	<10	NA	<10
Total bilirubin (mg/dL)	[0.2–1.2]	1.7	37.9	0.5	0.6	2.1	1.8	1.4	3.4	1.3	6.9	7.3	2.9	6.6	6.5	5.3
Indirect Bilirubin (mg/dL)	[0.2–1.0]	1.3	15.7	0.2	NA	1.8	1.2	0.5	2.6	0.9	6.7	6.8	2.8	5.4	6.3	4.8
Ferritin	[20–250: male, 10–80: female]	1612	2537.3	277.3	NA	3895	172	489	649.5	108.7	305.1	1663	NA	2583.0	NA	87.1
Membrane examination																
AGLT	[30 min < ]	NA	NA	NA	NA	NA	NA	NA	30 min<	30 min<	NA	NA	NA	NA	30 min<	30 min<
EMA (% of control)		114	NA	NA	89	103	NA	NA	113	97	112	111	113	112	99	93
FCM-OF (% of control)		193	NA	NA	219	122	NA	NA	132	143	112	113	186	179	142	125
Identified variants																
Genes		*PIEZO1*	*PIEZO1*	*PIEZO1*	*PIEZO1*	*PIEZO1*	*PIEZO1*	*PIEZO1*	*PIEZO1*	*PIEZO1*	*PIEZO1*	*PIEZO1*	*PIEZO1*	*PIEZO1*	*KCNN4*	*KCNN4*
RefSeq ID		NM_001142864.3	NM_001142864.3	NM_001142864.3	NM_001142864.3	NM_001142864.3	NM_001142864.3	NM_001142864.3	NM_001142864.3	NM_001142864.3	NM_001142864.3	NM_001142864.3	NM_001142864.3	NM_001142864.3	NM_002250.2	NM_002250.2
Affected exons		Exon 11	Exon 14	Exon 14	Exon 32	Exon 42	Exon 42	Exon 42	Exon 48	Exon 51	Exon 51	Exon 51	Exon 51	Exon 51	Exon 5	Exon 7
cDNA change		c.1281_1282ins[GG;1257_1281]	c.1792G > A	c.1792G > A	c.4370 C > T	c.6041 C > T	c.6041 C > T	c.6041 C > T	c.6968 A > C	c.7463 G > A	c.7483_7488dup	c.7483_7488dup	c.7483_7488dup	c.7483_7488dup	c.835 G > A	c.1055 G > A
Protein change		p.A427_L428insGMDQSYVCA	p.V598M	p.V598M	p.A1457V	p.T2014I	p.T2014I	p.T2014I	p.K2323T	p.R2488Q	p.L2495_E2496dup	p.L2495_E2496dup	p.L2495_E2496dup	p.L2495_E2496dup	p.A279T	p.R352H
Type		Insertion	Missense	Missense	Missense	Missense	Missense	Missense	Missense	Missense	Duplication	Duplication	Duplication	Duplication	Missense	Missense
Status		Hetero	Hetero	Hetero	Hetero	Hetero	Hetero	Hetero	Hetero	Hetero	Hetero	Hetero	Hetero	Hetero	Hetero	Hetero
dbSNP ID		−	−	−	rs532444891	−	−	−	−	rs749288233	rs1064793545	rs1064793546	rs1064793547	rs1064793548	−	rs774455945
SIFT (score)		NA	0.004	0.004	0.019	0.017	0.017	0.017	0.083	0	NA	NA	NA	NA	0.237	0
Polyphen2 (score)		NA	1	1	0.06	0.047	0.047	0.047	0.43	0.999	NA	NA	NA	NA	0.999	0.999
CADD_phred		NA	31	31	24.3	28.6	28.6	28.6	26.7	35	NA	NA	NA	NA	26	35
Clinvar		Not reported	Pathogenic/Likely pathogenic	Pathogenic/Likely pathogenic	Not reported	Not reported	Not reported	Not reported	Not reported	Not reported	Not reported	Not reported	Not reported	Not reported	Not reported	Pathogenic
ACMG criteria		PM2, PM4, PM6, PP4	PS1, PS3, PM2, PP3, PP4	PS1, PS3, PM2, PP3, PP4	PM2, PM6, PP3, PP4	PS4, PM2, PP3, PP4	PS4, PM2, PP3, PP4	PS4, PM2, PP3, PP4	PS4, PM2, PP3, PP4	PS1,PS3, PM2, PP1, PP3, PP4	PS1, PM2, PM4, PP1, PP3, PP4	PS1, PM2, PM4, PP1, PP3, PP4	PS1, PM2, PM4, PP1, PP3, PP4	PS1, PM2, PM4, PP1, PP3, PP4	PS1, PM5, PP2, PP3, PP4	PS1, PS3, PM6, PP3, PP4
Interpretation		Likely pathogenic	Pathogenic	Pathogenic	Likely pathogenic	Likely pathogenic	Likely pathogenic	Likely pathogenic	Likely pathogenic	Pathogenic	Pathogenic	Pathogenic	Pathogenic	Pathogenic	Likely pathogenic	Pathogenic
Reported/novel		Novel	Reported	Reported	Novel	Reported	Reported	Reported	Novel	Reported	Reported	Reported	Reported	Reported	Novel	Reported
Status of other polymorphic variants																
αLELY variants (c.5572 C > G)		−	Hetero	Homo	Hetero	−	NA	NA	−	−	−	Homo	Hetero	−	Hetero	−
αLELY variants (c.6531–12 C > T)		−	Hetero	Homo	Hetero	−	NA	NA	−	−	−	Homo	Hetero	−	Hetero	−
UGT1A1 variants (*6)		−	−	−	−	−	NA	NA	−	−	−	−	Hetero	−	−	−
UGT1A1 variants (*28)		−	−	−	Hetero	−	NA	NA	−	−	−	−	−	Hetero	−	Homo
Memphis I (SLC4A1: c.166 A > G)		−	−	−	−	−	NA	NA	Hetero	Homo	Hetero	−	−	Hetero	−	−
Memphis II (SLC4A1: c.2561 C > T)		−	−	−	−	−	NA	NA	−	Hetero	−	−	−	−	−	Hetero

*Pt* patient, *F* female, *M* male, *y* years, *m* months, *NA* not available, *[* *]* normal range, _ underbar indicates abnormal finding.

Ten families showed seven types of heterozygous *PIEZO1* variants (one insertion, five missense, and one in-frame duplication). Among them, two variants (p.A427_L428insGMDQSYVCA and p.K2323T) were novel and not included in the databases of ClinVar (https://www.ncbi.nlm.nih.gov/clinvar/) and gnomAD (http://www.gnomad-sg.org/). The p.A1457V variant identified in Patient 3 was included in the dbSNP database (https://www.ncbi.nlm.nih.gov/snp/) with ID = rs532444891. The minor allele frequency in the Japanese population was 0.00155, indicating that it is very rare in the general population. It is also identified in the Human Genetic Variation Database (https://www.hgvd.genome.med.kyoto-u.ac.jp/index.html), the variation database of the Japanese population, with a very low incidence^16^. Because no reports suggesting the p.A1457V variant as the disease-causing variant exists, we first report this as a novel disease-causing variant in this study.

Four variants (p.V598M, p.T2014I, p.R2488Q, p.L2495_E2496dup) were previously reported^6,8,17–19^. The p.V598M and p.T2014I were segregated in the families of Patients 2 and 4, respectively. The p.R2488Q variant is often reported as a disease-causing variant by many researchers and is registered in the dbSNP database with ID = rs749288233^6,18^. However, this has not been reported in ClinVar.

Heterozygous *KCNN4* variants were identified in two families. The variant p.R352H has been previously reported^9,20^. In *KCNN4*, p.R352H erythrocytes, preliminary data also suggested that altered channel activation kinetics led to erythrocyte dehydration^9^. Thus, we first report p.A279T as a novel disease-causing variant in this study.

## Discussion

In this study, 12 Japanese families suspected of having DHSt were genetically diagnosed with causative variants related to DHSt. Among them, 10 families (83%) had *PIEZO1* variants. Most *PIEZO1* variants were first identified in this study in Japanese patients.

Four of the ten families (40%) shared the recurrent variant (p.L2495_E2496dup). This variant was previously reported in a Japanese family associated with hereditary high phosphatidylcholine, hemolytic anemia, and hemochromatosis-induced diabetes mellitus^21^. The p.L2495_E2496dup is located at the junction of the α2 and α3 intracellular COOH-terminal domains, which is predicted to be involved in pore formation of the ion channel^22^. This variant causes changes in the hydropathicity profile in the regions where it is located, suggesting possible structural change^23^. Picard et al. described the clinical, hematologic and genetic characteristics of a retrospective series of 126 subjects from 64 families with DHSt^20^. Among them, 19 families showed *PIEZO1* variants, and 10 of 19 families (53%) had p.L2495_E2496dup. Although this variant has been reported frequently worldwide^8,18,20–24^, it is not included in ClinVar.

The other recurrent *PIEZO1* variants (p.V598M, p.T2014I, p.R2488Q) were first identified in the Japanese population, and two of them (p.V598M and p.T2014I) were confirmed to be segregated in the families; however, it is still unclear whether they came from the same founder. Further analyses would be needed.

*PIEZO1* encodes a mechanosensitive ion channel that translates a mechanical stimulus into calcium influx and is related to DHS1, which is a dominantly inherited red cell membrane disorder. No *PIEZO1* variant associated with loss-of-function (LoF) was found, and the pLI score of PIEZO1 was “0” in gnomAD. Therefore, *PIEZO1* is tolerant to LoF, and the gain-of-function mechanism is considered the mechanism rather than LoF, as mentioned above. Indeed, functional studies of DHS1-associated *PIEZO1* variants exhibited a partial gain-of-function phenotype, with many mutants demonstrating delayed channel inactivation^8^.

Although patients with DHSt often have fully or partially compensated hemolysis with few symptoms, iron overload is a universal finding, even in patients without transfusions or with only sporadic blood transfusions, and this causes progressive organ damage^25^. All patients with *PIEZO1* variations in this study showed elevated levels of ferritin, except a young 8-year-old patient. It was previously reported that ferritin levels at diagnosis were correlated with the age of patients^20^. The ferritin level of our patients also reflects this trend. Ma et al. showed that constitutive or macrophage expression of a gain-of-function Piezo1 allele in mice disrupts levels of the iron regulator hepcidin and causes iron overload^26^. They further show that *PIEZO1* is a key regulator of macrophage phagocytic activity and subsequent erythrocyte turnover^26^. Their discovery may be a new seed to treat hyperferritinemia in DHS1 patients.

In contrast, *KCNN4* encodes a Ca2 +-activated K+ channel. Although *KCNN4* has been reported to be associated with some diseases, including inflammatory bowel disease, Crohn’s disease, and Alzheimer’s disease, germline pathogenic variants in *KCNN4* have only been shown to be associated with DHS2^27–30^. To date, ten *KCNN4* variants (p.V222L, p.V282M, p.V282E, p.S314P, p.A322V, p.H328R, p.H340R, p.H340N, p.R352H, p.V369_Lys373del) have been reported in patients with DHS2 ^9,18,20,24,31,32^. Among them, p.R352H identified in patient 12 in our study has been recurrently identified^20^. The novel p.A279T, first identified in our study, is located near p.V282M and p.V282E. The 231-289^th^ amino acids of *KCNN4* form a two-pore potassium channel domain, and the 304-377^th^ amino acids form a calmodulin-binding domain^24,33^. These regions are highly conserved and have an important role in *KCNN4*.

In patients’ RBCs that carry *KCNN4* variations, the channel conductance is considered to be increased. Despite leading to a more active channel, the gain-of-function in *KCNN4* is not systematically linked to RBC dehydration, and routine hematological tests have failed to clearly diagnose DHSt^20^. Rivera et al. analyzed the characteristics of two de novo *KCNN4* variants (p.V222L and p.H340N)^32^. However, the data did not correlate with RBC dehydration caused by *KCNN4* gain-of-function, raising the question of whether this pathology should be classified as a DHSt. Moreover, it emphasized the difficulty of diagnosing altered RBC permeability facing *KCNN4* variants and the great variability in RBC phenotypes associated with the *KCNN4* gain-of-function mechanism^32^.

It is important to distinguish DHSt from hereditary spherocytosis (HS). Splenectomy should be avoided in patients with DHSt because it seems to aggravate the risk of thrombosis^25^. Especially in DHS1, postsplenectomy thrombotic events are called a major risk^20^. In our study, thrombotic events were frequent in splenectomized Patient 2 with DHS1. On the other hand, no thrombotic events occurred in splenectomized Patient 14 with DHS2. Picard et al. also reported that none of the four DHS2 splenectomized patients experienced thrombosis^20^. To date, the number of reported cases of DHS2 is lower than that for DHS1. However, we should not allow definitive conclusions to be drawn on this issue. Further evaluation of case information is needed, and patients splenectomized before being genetically diagnosed should be carefully monitored for a long time.

In this study, two patients (Patient 2 and Patient 3) with DHS1 had a history of fetal edema. A previous report showed that perinatal edema was observed in DHS1 but not in DHS2 patients^20^. The severity of perinatal edema is heterogeneous, so careful pregnancy follow-up with ultrasound monitoring is needed in both genotypes^34^.

We performed functional examinations of the RBC membrane. It is known that the EMA test and FCM-OF test are good combinations for the diagnosis of HS^35^. Recently, the results of these tests for DHSt patients were reported. Zama et al. showed that the result of the EMA test of DHSt patients is normal or increased^36^. Our data are consistent with their results.

In DHSt, a dysfunctional membrane protein eventually leads to potassium leakage out of the RBCs that exceeds the inward flux of sodium, and the accompanying net loss of water results in RBC dehydration, shrinkage, fragility, and hemolysis^25^. The results of the FCM-OF test in our patients reflect this pathology.

Although we genotyped known polymorphisms of αLELY, *UGT1A1*, and Memphis I and II, we could not identify any correlation between clinical findings and severity.

Recently, *ABCB6* has been reported to be responsible for familial pseudohyperkalemia, a disorder related to DHSt^37^. However, this gene was not included in the gene panel used in this study. Therefore, it is the subject of our future project to investigate whether *ABCB6* is related to patients with DHSt without disease-causing variants of *PIEZO1* and *KCNN4*.

In conclusion, here, we first report the hematological, clinical, and genetic features of DHSt in Japan. Comprehensive genomic analysis is a powerful tool for understanding the genetic cause of congenital hemolytic anemia and would be beneficial for the molecular diagnosis and clinical management of DHSt.

### Supplementary information

Image of the elecropherogram of Sanger sequencing

Images of the elecropherograms of Sanger sequencing

## References

[1] Andolfo I, Russo R, Gambale A, Iolascon A (2016). New insights on hereditary erythrocyte membrane defects. Haematologica.

[2] Narla J, Mohandas N (2017). Red cell membrane disorders. Int. J. Lab. Hematol..

[3] Vives-Corrons JL, Krishnevskaya E, Rodriguez IH, Ancochea A (2021). Characterization of hereditary red blood cell membranopathies using combined targeted next-generation sequencing and osmotic gradient ektacytometry. Int. J. Hematol..

[4] Mohandas N (2018). Inherited hemolytic anemia: a possessive beginner’s guide. Hematol. Am. Soc. Hematol. Educ. Program..

[5] Iolascon A, Andolfo I, Russo R (2019). Advances in understanding the pathogenesis of red cell membrane disorders. Br. J. Haematol..

[6] Andolfo I (2013). Multiple clinical forms of dehydrated hereditary stomatocytosis arise from mutations in PIEZO1. Blood.

[7] Zarychanski R (2012). Mutations in the mechanotransduction protein PIEZO1 are associated with hereditary xerocytosis. Blood.

[8] Albuisson J (2013). Dehydrated hereditary stomatocytosis linked to gain-of-function mutations in mechanically activated PIEZO1 ion channels. Nat. Commun..

[9] Rapetti-Mauss R (2015). A mutation in the Gardos channel is associated with hereditary xerocytosis. Blood.

[10] Shimojima Yamamoto K (2022). Clinical and genetic diagnosis of thirteen Japanese patients with hereditary spherocytosis. Hum. Genome Var..

[11] Richards S (2015). Standards and guidelines for the interpretation of sequence variants: a joint consensus recommendation of the American College of Medical Genetics and Genomics and the Association for Molecular Pathology. Genet Med..

[12] Delaunay J (2004). Different impacts of alleles alphaLEPRA and alphaLELY as assessed versus a novel, virtually null allele of the SPTA1 gene in trans. Br. J. Haematol..

[13] Kaliniczenko, A. et al. Frequency of the DI*A, DI*B and Band 3 Memphis polymorphism among distinct groups in Brazil. *Hematol. Transfus. Cell. Ther.* **S2531–1379**, 00052–00059 (2022). 10.1016/j.htct.2022.03.002 PMC10433289 35662509

[14] Rets A, Clayton AL, Christensen RD, Agarwal AM (2019). Molecular diagnostic update in hereditary hemolytic anemia and neonatal hyperbilirubinemia. Int. J. Lab. Hematol..

[15] Wilmotte R, Marechal J, Delaunay J (1999). Mutation at position -12 of intron 45 (c–>t) plays a prevalent role in the partial skipping of exon 46 from the transcript of allele alphaLELY in erythroid cells. Br. J. Haematol..

[16] Higasa K (2016). Human genetic variation database, a reference database of genetic variations in the Japanese population. J. Hum. Genet..

[17] Andolfo I (2018). PIEZO1-R1864H rare variant accounts for a genetic phenotype-modifier role in dehydrated hereditary stomatocytosis. Haematologica.

[18] Glogowska E (2017). Novel mechanisms of PIEZO1 dysfunction in hereditary xerocytosis. Blood.

[19] Gnanasambandam R (2018). Increased red cell KCNN4 activity in sporadic hereditary xerocytosis associated with enhanced single channel pressure sensitivity of PIEZO1 mutant V598M. Hemasphere.

[20] Picard V (2019). Clinical and biological features in PIEZO1-hereditary xerocytosis and Gardos channelopathy: a retrospective series of 126 patients. Haematologica.

[21] Imashuku S (2016). PIEZO1 gene mutation in a Japanese family with hereditary high phosphatidylcholine hemolytic anemia and hemochromatosis-induced diabetes mellitus. Int. J. Hematol..

[22] More TA, Dongerdiye R, Devendra R, Warang PP, Kedar PS (2020). Mechanosensitive Piezo1 ion channel protein (PIEZO1 gene): update and extended mutation analysis of hereditary xerocytosis in India. Ann. Hematol..

[23] de Meira Oliveira P (2020). Heterogeneous phenotype of Hereditary Xerocytosis in association with PIEZO1 variants. Blood Cells Mol. Dis..

[24] Fermo E (2017). Hereditary xerocytosis due to mutations in PIEZO1 gene associated with heterozygous pyruvate kinase deficiency and beta-thalassemia trait in two unrelated families. Case Rep. Hematol..

[25] Frederiksen H (2019). Dehydrated hereditary stomatocytosis: clinical perspectives. J. Blood Med..

[26] Ma S (2021). A role of PIEZO1 in iron metabolism in mice and humans. Cell.

[27] Süss C (2020). KCNN4 expression is elevated in inflammatory bowel disease: this might be a novel marker and therapeutic option targeting potassium channels. J. Gastrointestin. Liver Dis..

[28] Simms LA (2010). KCNN4 gene variant is associated with ileal Crohn’s disease in the Australian and New Zealand population. Am. J. Gastroenterol..

[29] Kosoy R (2022). Genetics of the human microglia regulome refines Alzheimer’s disease risk loci. Nat. Genet..

[30] Philp AR (2018). Kcnn4 is a modifier gene of intestinal cystic fibrosis preventing lethality in the Cftr-F508del mouse. Sci. Rep..

[31] Mansour-Hendili L (2021). Multiple thrombosis in a patient with Gardos channelopathy and a new KCNN4 mutation. Am. J. Hematol..

[32] Allegrini B (2022). New KCNN4 variants associated with anemia: stomatocytosis without erythrocyte dehydration. Front. Physiol..

[33] Rivera A (2022). The erythroid K-Cl cotransport inhibitor [(dihydroindenyl)oxy]acetic acid blocks erythroid Ca(2+)-activated K(+) channel KCNN4. Am. J. Physiol. Cell Physiol..

[34] Beneteau C (2014). Recurrent mutation in the PIEZO1 gene in two families of hereditary xerocytosis with fetal hydrops. Clin. Genet..

[35] Arora RD (2018). Flow cytometric osmotic fragility test and eosin-5’-maleimide dye-binding tests are better than conventional osmotic fragility tests for the diagnosis of hereditary spherocytosis. Int. J. Lab. Hematol..

[36] Zama D (2020). A novel PIEZO1 mutation in a patient with dehydrated hereditary stomatocytosis: a case report and a brief review of literature. Ital. J. Pediatr..

[37] Andolfo I (2013). Missense mutations in the ABCB6 transporter cause dominant familial pseudohyperkalemia. Am. J. Hematol..

