# Supplementary material for: Variant spectrum of PIEZO1 and KCNN4 in Japanese patients with dehydrated hereditary stomatocytosis
Source: Hum Genome Var. 2023 Mar 2;10:8. doi: 10.1038/s41439-023-00235-y (PMC9981561; doi:10.1038/s41439-023-00235-y)
Supplement: Supplementary file 1 — Image of the elecropherogram of Sanger sequencing [file 41439_2023_235_MOESM1_ESM.pdf]

Supplemental Figure S1. Image of the electropherogram of Sanger sequencing

Patient 1: p.A427_L428insGMDQSYVCA

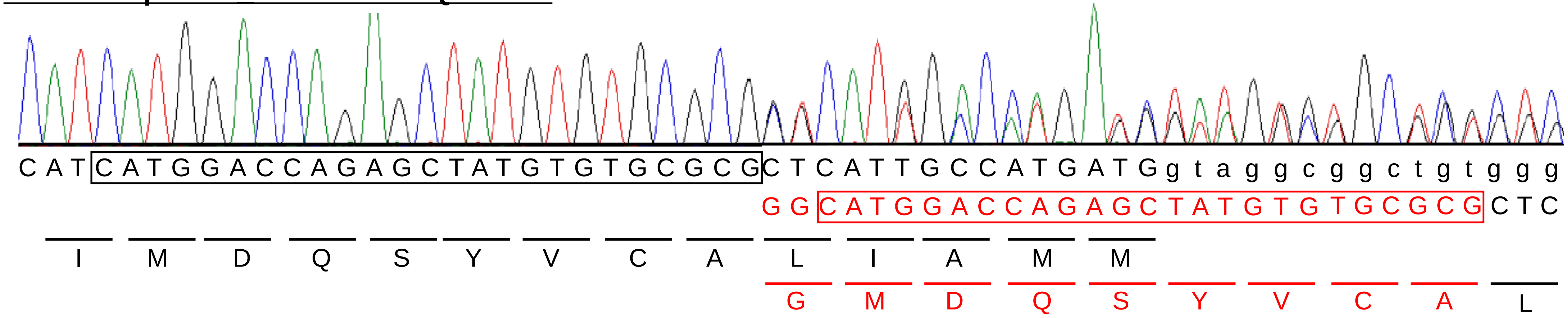

Red characters indicate the 27-bp insertion, which is consisted with the 25-bp duplication in addition with GG. Black and red rectangles indicate the original and duplicated 25-bp nucleotide segments, respectively. By adding "GG", the inserted 25-bp nucleotide segment caused 9 amino-acid insertion reading to in-flame insertion.
