# Supplementary material for: Variant spectrum of PIEZO1 and KCNN4 in Japanese patients with dehydrated hereditary stomatocytosis
Source: Hum Genome Var. 2023 Mar 2;10:8. doi: 10.1038/s41439-023-00235-y (PMC9981561; doi:10.1038/s41439-023-00235-y)

Supplemental Figure S2. Images of the electropherograms of Sanger sequencing

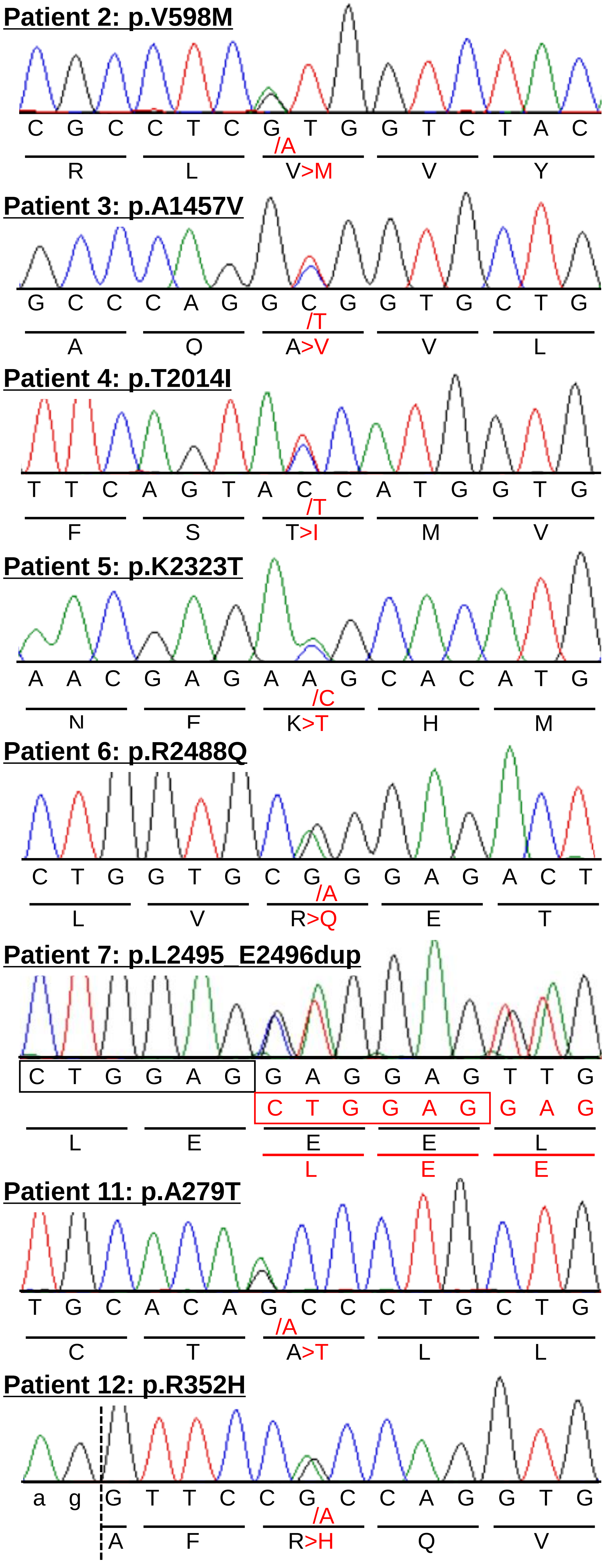

Supplement: Supplementary file 2 — Images of the elecropherograms of Sanger sequencing [file 41439_2023_235_MOESM2_ESM.pdf]
